# Intravenous immunoglobulin for inflammatory demyelinating polyneuropathy

**DOI:** 10.1007/s00415-021-10908-9

**Published:** 2021-12-08

**Authors:** M. Talaei, N. P. Robertson

**Affiliations:** 1 Department of Neurology, University Hospital of Wales, Heath Park, Cardiff, CF14 4XN UK; 2 Division of Psychological Medicine and Clinical Neuroscience, Department of Neurology, University Hospital of Wales, Cardiff University, Heath Park, Cardiff, CF14 4XN UK

Intravenous immunoglobulin (IVIg) plays a key role in treatment of inflammatory demyelinating polyneuropathies such as Guillain–Barre syndrome (GBS). However, individual response to treatment can be unpredictable, and for those most severely affected, a second dose of IVIg (SIV) is commonly administered despite limited evidence for efficacy. In this month’s journal club, we review two studies which explore the efficacy of second dosing and a third study exploring neurophysiological factors predicting relapse in chronic inflammatory polyneuropathy.

## Original research: Second IVIg course in GBS with poor prognosis: the non-randomised ISID study

ISID was a non-randomised International Second IVIg dose comparing disease course in poor prognosis GBS patients treated with one or two courses of IVIg to determine whether SID improved functional outcome. Data were collected from a prospective observational International GBS Outcome Study (IGOS). Modified Erasmus GBS Outcome Score (MEGOS) at week 1 was used to predict patients with poor prognosis. Primary endpoint was improved functional outcome on the GBS disability scale after 4 weeks. Secondary endpoints included GBS disability score at 26 weeks, ability to walk independently at 26 weeks, ventilatory requirement, ITU admission, and GBS-related mortality at 6 months.

260 (32%) of 807 eligible patients were considered to have poor prognosis and 237 were included (33 patients excluded as enrolled in other trials or received IVIG for treatment fluctuation). 199 patients received one course of IVIG (controls), 20 patients received an early second IVIG (within 1–2 weeks of initial course), and 18 received a late second IVIG course (2–4 weeks after initial course). Of the 199 control patients 160 (80%) received only IVIG in the first 4 weeks, 32 received IVIG and plasma exchange, and 7 had further IVIG course after 4 weeks, and from each two later groups, one patient eventually had alternative diagnosis. From the 20 early second IVIG group, 16 (80%) had two courses of IVIG and the remaining 4 had mixed IVIG and plasma exchange. From the 18 late second IVIG group, 14 (78%) patients were only treated with two IVIG and 4 also had plasma exchange. 167/199 (84%) of control group, 18/20 (90%) of early IVIG group, and 16/18 (90%) of late IVIG group had primary endpoint assessment.

Ventilatory support was different at study entry between control (*n* = 36, 18%), early IVIg (*n* = 9, 45%), and late IVIg group (*n* = 6, 33%) (three-way p value, *p* = 0.01).

There was no significant difference in GBS disability score 4 weeks after study entry (adjusted OR 0.70 (95% CI 0.16–3.04) for early IVIg group vs 0.66 (95% CI 0.18–2.50) for late IVIG group). There was no significant difference in GBS disability score at 26 weeks [adjusted OR 0.89 for the early group (95% CI 0.22–3.53) vs 0.40 (95% CI 0.10–1.62) for the late group]. ICU admission was longest in patients with late IVIG (64 days), compared to controls and early IVIG group (30 vs 31 days). Patients in the late group required longer ventilatory support (76 days) than controls and early group (27 vs 55 days). Serious complications of IVIG were not reported. Nine (6%) of control patients vs none in the second IVIG group died within 6 months.

**Comments:** This study provides no evidence for efficacy of second IVIg in patients with GBS with poor prognosis. However, the observational nature of the study and measuring the functional status only at 4 weeks result in significant limitations. Also, the number of patients in the second IVIG groups was small (20 and 18). Definite evidence for the value of second dosing requires evidence from a prospective randomised trial.

Verboon C, et al. *J Neurol Neurosurg Psychiatry* 2020;91:113–121.

## Second IVIG dose in patients with GBS with poor prognosis (SID-GBS): a double-blind, randomised, placebo-controlled trial

This was a double-blind, randomised, placebo-controlled phase 3 trial across 59 hospitals in the Netherlands. GBS patients aged 12 years or older who required IVIG were eligible for recruitment (327 patients). Patients were randomly allocated (1:1) to receive SID or placebo for 5 days, at 7–9 days after the start of the first standard IVIG treatment (2 g/kg for 5 days). Modified Erasmus GBS Outcome Scale (MEGOS) 7–9 days after start of the standard IVIG was used to identify patients with poor prognosis and patient with MEGOS of six or more was randomly assigned to SID or placebo.

Of 327 patients, 112 were predicted to have poor prognosis; 13 were excluded as a result of declined consent or not meeting inclusion criteria. 99 patients were randomised (53 to SID and 46 to placebo). 49 patients from SID group and 44 patients from placebo group were analysed in the modified intention-to-treat analysis. Primary endpoint was improved functional outcome on the GBS disability scale after 4 weeks. A 20% difference in the proportion of patients improving at least one grade on GBS disability scale considered clinically relevant. Secondary outcomes were assessed at weeks 4, 8, 12, and 26, and comprised the GBS disability scale, MRC sum score, Overall Neuropathy Limitations Scale, ventilation support, ICU admission, mortality, and serum IgG concentrations at subsequent timepoints.

The adjusted common odds ratio for improvement on the GBS disability score at 4 weeks was 1·4 (95% CI 0·6–3·3; *p* = 0·45) VS unadjusted common odds ratio 1·3 (95% CI 0·6–3·3). There was no difference between treatment groups for any of the secondary outcomes including GBS disability scores at weeks 8, 12, and 26, improving one grade or more on the GBS disability scale at four different time points, MRC sum score and Overall Neuropathy Limitations Scale, duration of hospital admission, ICU admission, and mechanical ventilation. Four patients died during the trial, all from the SID group. Serious adverse events, including thromboembolic events, also occurred more often in the SID group than the placebo (51% *vs* 23%).

**Comments:** IVIG in GBS appears to be predominantly effective early in the disease course and there was no significant clinical benefit of a second IVIG course. Furthermore, patients who were given SID experienced a greater frequency of severe adverse events, including death and thromboembolism, compared to the single IVIG dose group and placebo, indicating significant iatrogenic risks.

Walgaard C, et al. *Lancet Neurol*. 2021;20(4):275–283.

## Electrophysiological testing in CIDP patients treated with subcutaneous immunoglobulin: the polyneuropathy and treatment with Hizentra (PATH) study

Electrophysiology has prognostic value in CIDP by defining a number of features used to predict relapse after IVIG withdrawal. This study examined relapse rates in IVIG-dependent CIDP patients randomised to either low (0.2 g/kg) or high (0.4 g/kg) dose weekly subcutaneous immunoglobulin (SCIG; IgPro20, Hizentra) or placebo and showed reduced relapse rates with both IgPro20 doses versus placebo.

Patients were aged 18 or older with a definite or probable CIDP diagnosis according to EFNS/PNS 2010 criteria and responded to IVIG treatment within 8 weeks prior to enrolment. All eligible subjects progressed through three study periods: an IgG withdrawal period (up to 12 weeks), an IVIG re-stabilisation period (up to 13 weeks), and an IgPro20 SCIG treatment period (24 weeks). Electrophysiology was performed at the start and end of the subcutaneous treatment interval (at the week 25 visit) or at early termination. Three motor nerves (median, ulnar, and peroneal) were measured. All studies performed using surface stimulating and recording electrodes, under careful temperature control; parameters measured included distal and proximal motor latencies, distal and proximal CMAP amplitudes, and motor nerve conduction velocities.

Primary outcome was the percentage of subjects with CIDP relapse (defined as ≥ 1 point deterioration in INCAT score) or withdrawal for any reason during the 24-week IgPro20 SCIG treatment period. The secondary outcomes were measured with clinical scales (grip strength, MRC sum score, Inflammatory Rasch-built Overall Disability Scale), quality of life, satisfaction questionnaires, and electrophysiology.

172 patients were randomised to IgPro20 or placebo. 57 patients were assigned to 0.2 g/kg IgPro20, 58 patients to 0.4 g/kg IgPro20, and 57 patients were assigned to placebo. On average, placebo patients were on treatment 4 weeks less than active treatment groups in the study due to earlier deterioration and withdrawal from the study. There was a significant increase in average proximal latency in placebo group (+ 1.1 ms) indicating electrophysiologic deterioration vs stable feature in IgPro20 (0.2 g/kg + 0.1 ms; 0.4 g/kg − 0.1 ms). Distal latencies were also more prolonged in placebo versus IgPro20 (+ 0.4 ms in placebo, + 0.1 ms in 0.2 g/kg IgPro20 group, and no change in 0.4 g/kg IgPro20 group). Average motor nerve conduction velocity decreased noticeably in placebo (− 1.6 m/s) and increased in both IgPro20 groups (+ 0.2 m/s and + 1.0 m/s). There was no significant change in conduction block and CMAP amplitudes.

**Comments:** This study supports the effectiveness of maintenance IgPro20. The study was not powered to show changes in electrophysiology and the data were used as exploratory parameters. Some patients did not have electrophysiology at baseline or endpoint and the study interval was short. The value of small changes in electrophysiology seen as deterioration of speed of conduction observed in placebo group is uncertain and can only be inferred from other studies.

Vera B, et al. *Clin Neurophysiol*, 132 (2021): 226–231.

